# Supplementary material for: Are carbon emissions trading and green financial instruments synergistic? -Comprehensive quantitative research based on content analysis
Source: PLoS One. 2024 Mar 7;19(3):e0298601. doi: 10.1371/journal.pone.0298601 (PMC10919601; doi:10.1371/journal.pone.0298601)
Supplement: S1 Data — (ZIP) [file pone.0298601.s001.zip › Data/地级市Z_F工作报告.zip!/╔╜╬≈/┬└┴║╩╨/└┤╫╘╣1⁄2╓┌║┼í╛┬φ┐╦╩2╛▌═°í┐_2010.docx]

各位代表：

现在我代表市人民政府向大会作报告，请予审议，并请各位市政协委员和其他列席人员提出意见。

一、2009年工作回顾

2009年是吕梁经济发展进程中极不平凡的一年。受国际金融危机的严重冲击，加之长期积累的体制性、结构性矛盾的集中显现，市域经济持续下滑，企业普遍经营困难，财政收支矛盾加剧。面对严峻挑战，我们在中共吕梁市委的坚强领导下在市人大、市政协的监督支持下，团结带领全市人民，按照“三三战略”总体部署，以保增长、保民生、保稳定为主线，以调结构、促改革、强基础为支撑，坚定信心，团结拼搏，攻坚克难，积极应对，在逆境中保持了全市经济社会健康平稳发展的良好势头。地区生产总值完成611．3亿元，同比增长2．5％；全社会固定资产投资完成388．8亿元，增长3．5％；工业增加值完成343．7亿元，增长0．1％；财政总收入、一般预算收入分别完成165．4亿元、59．7亿元，增长0．6％、8．4％；社会消费品零售总额完成197亿元，增长22．4％；城镇居民人均可支配收入达到13795元，增长8％；农民人均纯收入达到3426元，增长7．1％；居民消费价格总水平下降0．06；城镇登记失业率控制在3％以内。以上主要经济指标的完成情况，纵向看好于亚洲金融危机时期，横向看高于部分周边地市，且发展态势逐月逐季向好，有的已经提前实现了“十一五”规划目标。在去年这样复杂而困难的形势下，这是十分难得的，也是令人鼓舞的。

一年来，我们主要做了以下工作：

1、千方百计保增长，全力推动经济平稳较快发展全面落实扩大内需政策。面对市域经济严重下滑的趋势，我们始终把保持经济平稳较快增长作为经济工作的首要任务，认真落实党中央、国务院和省委、省政府的决策部署，千方百计扩大投资、拉动内需，先后争取总投资21．36亿元的中央扩内需投资项目四批、690项，完成投资11．1亿元，已完工491项。

扎实开展企业帮扶工作。面对普遍出现的企业运行困难，我们及时提出“保企业就是保增长”的思路，严格执行国家税收“三免三减半”、社会保障“五缓四降一补”等政策，制定实施了领导干部包联企业等一系列制度措施，促成158户重点企业与金融机构合作，一次签约金额174．4亿元。孝义楼东公司成功上市，交城宏特、离石大土河、离柳焦煤等企业上市前期工作进展顺利。

全力推进重点工程建设。争取省级重点工程25项，确定市级重点工程23项，省、市两级重点工程开工率分别达到72％、96％。吕梁民用机场建设进展顺利、山西中南部铁路大通道开工前期准备就绪，太中银铁路（吕梁段）年内即可通车，太佳高速、汾平高速分别完成投资总额的70％、60％，国省道干线公路和“通达”、“通畅”工程建设任务顺利完成。柏叶口水库包括龙门渠项目、坪底水库正式开工，横泉水库供水工程竣工通水，沿黄提灌工程即将实施，新建淤地坝98座。中阳500千伏输变电工程建成投运，兴县500千伏输变电工程完成选址，一批220千伏、110千伏输变电工程相继竣工。

2、多措并举调结构，着力推动经济发展方式转变

不断提升主导产业素质。积极稳妥地开展煤矿企业兼并重组整合并取得阶段性成果。全市矿井总数（不包括省属企业原有矿井）由355处压减为111处，年产能由8557万吨提升到10380万吨，办矿主体由170个减少到31个，其中民营主体占到73．4％。整合矿井签约率、接管率、主体企业资金到位率、采矿证申领上报率均达100％，全市原煤月生产能力已恢复到670万吨。煤炭这一引领吕梁经济发展的龙头产业步入全新发展阶段。积极推动焦化、冶金、电力等传统产业布局调整、产能置换等工作，全面启动杏花村酒业集中发展区建设，大力推动循环经济基地和园区建设，新型工业化率提高到39％，工业废渣、废气回收利用率分别达到57％、65％。全面推进省、市服务业发展“1＋10”重点项目，第三产业的比重上升到30．15％，比上年增加5．15个百分点。

着力夯实“三农”发展基础。认真落实强农惠农政策，粮食、农资等补贴资金兑现2．35亿元，增长33．5％。推动农业产业布局优化，粮食产量达8．94亿公斤，为新世纪以来最好收成。新培育市级以上龙头企业71户，龙头企业销售收入达16．8亿元。农村经济总收入372．6亿元，增长3％。深入实施“三大经济林覆盖”工程，新栽植干果经济林40万亩，完成造林绿化75．4万亩。顺利实现新农村“四化四改”、“五个一工程”、“五四工程”年度建设目标。投入扶贫资金14．35亿元，新解决12万绝对贫困人口的温饱问题。新转移农村劳动力5．12万人，农民人均工资性收入达2305元，增加62元。

积极实施科技推广与创新。深入开展科技示范创建工作，汾阳、文水被列入全国科技富民强县计划。引进新技术、新成果67项，推广实用技术2000余件。科技节能型减排达标企业达35户，4户被列入省科技创新型试点企业。实施各类科技项目148项，8项科技成果达到国内先进水平，4项进入国际先进行列。

深入推进节能减排工作。以“工业节能、源头治污”为重点，持续实施环境保护和节能降耗攻坚战，坚决淘汰“两高一资”项目及落后产能，严厉打击土小污染企业反弹。万元地区生产总值综合能耗下降5．66％；二氧化硫、化学需氧量分别减排8．1％、6．8％，市区大气综合污染指数为1．86，下降8．86％；全市13个县（市、区）环境空气质量二级以上天数达到4259天，同比增加447天，市区达到359天，继续保持全省第一。

3、全力以赴保民生，着力加快和谐社会建设步伐

扎实推进重点民生工程。在财力紧张的条件下，千方百计压减一般性支出，大幅增加民生工程支出。新建经济适用房和廉租房6129套、36．24万平方米，建成棚户区改造和采煤沉陷区治理住房12990套、110．2万平方米。改造校舍危房面积76．49万平方米，提前一年完成522个空白村卫生所建设任务，完成697个行政村、4624．5公里的村通公路工程，新增农村饮水安全工程覆盖人口37．95万人，新增110个村通广播电视。市政府向全市人民承诺的“五个全覆盖”年度任务全面完成，“十件实事”全部兑现！

继续加快社会事业发展。进一步完善义务教育经费保障机制，标准化建设和“双百”示范校建设稳步推进；普通高中办学水平整体提升，中职教育、成人教育协调发展；中小学教师队伍建设取得明显成效；吕梁学院主体工程完工并顺利通过升本评估验收。深入推进医药卫生体制改革，启动基本药物制度试点，公共卫生体系进一步完善，县乡医疗卫生机构达标率达91．1％。大力开展以防“甲流”为重点的疫病防控工作，深入推进食品、药品综合整治，人民群众健康安全得到有力保障。全面完成文化体制改革，进一步加强广播电视、新闻出版、非物质文化遗产保护等工作。人口和计生工作继续保持全省先进。全民健身运动和竞技体育再上新台阶。妇女儿童、劳动竞赛、青少年事务、民族宗教、外事侨务、档案史志、防震、气象、老龄、残疾人、人民武装等工作都取得了新的成绩。

不断强化社会保障体系建设。全面启动城镇居民医疗保险，扎实开展新型农村养老保险试点工作。逐步完善城乡医疗救助制度，继续提高农村五保供养水平、优抚对象补助标准与城乡低保保障水平，积极支持社会福利和慈善事业发展。新型农村合作医疗参合农民达到262万人，社会保险综合覆盖率达到82％；城市低保实现了动态管理下的应保尽保。继续加强就业再就业工作，积极开辟高校毕业生创业就业渠道，新增城镇就业3．4万人，城镇登记失业率控制在3％以下。

4、坚持不懈保稳定，着力改善经济社会发展环境

切实加强安全生产工作。深入开展安全生产专项整治，安全隐患与非法违法生产行为得到有效治理。认真落实安全生产十项制度，进一步强化政府、部门、企业安全责任，以“一岗双责”、“三委派”人员、“五人监管小组”为重点的安全责任制度体系不断完善。严肃对待事故，严格责任追究，全年查处各类安全责任事故16起，77人受到党纪政纪处分。全市安全生产事故起数、死亡人数同比分别下降0．8％、14％，其中煤矿安全生产事故起数、死亡人数同比分别下降71％、95％，未发生一次死亡10人以上事故，安全生产形势明显好转。

不断强化社会管理。继续完善社会治安防控以及公共应急管理制度、机制，进一步提高处置突发性公共事件的能力，圆满完成国庆60周年安保任务。国防后备力量建设成效显著。积极开展抗雪救灾行动，免费救助滞留人员5万余人次。信访工作再上新台阶，“信访积案化解年”活动取得积极成效。进一步加强社会综治、打黑除恶等工作，有力保障了全市政治稳定、社会安定。

5、持续推进改革开放和政府自身建设，着力增强经济社会发展活力和动力

不断加快改革开放步伐。全面启动政府机构改革、事业单位分类改革和事业单位人员聘用、岗位设置、收入分配等改革。增值税转型改革落实到位。集体林权制度改革试点任务全面完成。深入推进投融资体制改革，农村小额贷款组织规范发展，中信银行、民生银行落户吕梁。率先组建市级农业担保公司和创业投资公司，“三农”贷款担保和中小企业融资平台建设实现重大突破。招商引资和对外经济合作逆势上扬，全年实际利用市外资金1．79亿美元，居全省前列。

继续加强政府自身建设。主动接受人大和政协的监督，积极支持各民主党派、无党派人士参政议政，办理人大代表、政协委员的意见、建议673件。深入开展“五五”普法，认真落实依法治市措施，严格执行行政执法责任制、执法过错追究制等制度，积极推进依法行政。修订完善市政府《工作规则》，“按规律办事、按规定办事、按规矩办事”渐成氛围。认真落实党风廉政建设责任制，深入开展煤焦领域反腐败专项斗争，制定并实施了规范煤炭税费征管、加强工程建设项目监管等规章制度，源头防腐力度进一步加大。

各位代表，回顾过去一年的工作，我们在空前复杂、异常困难的形势下，积极应对国际金融危机的冲击，取得这些成绩实属不易。这是市委统揽全局、科学决策的结果，是四大班子风雨同舟、团结奋斗的结果，是全市人民顽强拼搏、无私奉献的结果。在此，我代表市人民政府，向各位人大代表、政协委员，向各民主党派、人民团体和各界人士，向驻吕解放军指战员、武警官兵和公安政法干警，向全市人民及所有关心支持吕梁发展的朋友，表示衷心的感谢，并致以崇高的敬意！

在肯定成绩的同时，我们也清醒地认识到发展中仍存在不少困难和问题。主要是：产业结构升级、发展方式转变、市域经济低碳化发展的基础有待继续加强；安全生产、安全发展面临的新情况、新挑战亟需科学应对；统筹城乡和经济社会发展的任务还非常艰巨；经济社会发展对公共财政需求大与地方可用财力增长慢的矛盾仍十分突出；政府职能转变、作风建设和新形势新任务的要求还有一定差距。对此，我们将高度重视，采取更加有效的措施认真加以解决。

二、2010年主要任务

今年是实现“十一五”规划目标的决战之年，是启动“十二五”发展规划的基础之年。做好今年的政府工作，意义重大。今年政府工作的总体要求是：以科学发展观为统领，全面贯彻落实市委二届六次全委（扩大）会议精神，紧紧围绕“三三战略”的总体部署，以保持经济平稳较快发展和加快转变发展方式为重点，全面实施新一轮重点项目攻坚，着力夯实“三农”发展基础，继续加快经济结构战略性调整，积极探索经济发展低碳化路径，加强城乡建设和城市管理，推进教育振兴和科技创新，强化社会建设和民生工作，切实维护社会和谐稳定，不断加快富裕、生态、特色新吕梁建设进程。

综合考虑各方面的因素，今年全市经济社会发展主要预期目标是：地区生产总值增长8％，全社会固定资产投资增长23．5％，工业增加值增长16．4％，财政总收入和一般预算收入分别增长8．8％、13．8％，社会消费品零售总额增长16．8％，城镇居民人均可支配收入、农民人均纯收入分别增长9％、10％，居民消费价格涨幅控制在3％以内。主要约束性指标是：万元生产总值综合能耗下降4．6％，二氧化硫、化学需氧量排放总量控制在8．4万吨、3．08万吨之内，大气综合污染指数控制在1．9以内，城镇新增就业岗位3万个，城镇登记失业率控制在3％以内，人口自然增长率控制在6‰以内。

完成上述目标，必须紧紧围绕市委二届六次全委（扩大）会议提出的“实施七大系统工程，谋求七个全新突破”的经济工作总体思路，在统筹推进的同时，重点做好以下七方面的工作：

1、全面启动“343”项目建设，夯实经济平稳较快发展基础

高标准确定攻坚项目。适应国家经济政策适度调整和淘汰落后产能的形势变化，坚持产业开发与基础设施建设并重、强市与惠民并进、国有投资与民间投资并举，突出“绿色、循环、低碳”三大特征，集中3年时间重点推进486个总投资3013亿元的项目建设（简称“343”项目）。一是抓好119个总投资587．4亿元的煤炭提升项目，改扩建、新建一批以综采为主的机械化矿井，提高产业集中度；二是抓好125个总投资1113．2亿元的工业转型项目，以大型焦化、冶炼、电力、化工、制造项目为重点，切实加快新型能源化工基地建设步伐；三是抓好81个总投资86．2亿元的农业龙头项目，以龙头企业为引擎，辐射带动特色农业和农副产品加工业快速发展；四是抓好50个总投资108．9亿元的三产牵动项目，加快建设一批以物流、商贸、酒店餐饮、旅游为重点的产业项目，努力使服务业发展水平与工业化水平相适应；五是抓好111个总投资1117．2亿元的基础设施及惠民项目，着力缓解基础设施、社会事业滞后的问题。

宽视野拓展融资渠道。着力打造政府融资新平台，逐步规范城投公司运行，筹备组建水投公司，充分发挥各级财政贷款贴息、项目启动资金的拉动效应，让政府投资带动多倍数的社会投资。强化项目与政策的对接，最大限度争取上级支持。着眼优化结构、提升区域经济整体竞争力，多渠道吸引市外资金和客商进入我市。鼓励各级政府与金融投资机构合作，拓宽间接融资渠道。引进战略投资者组建股份制公司，积极推进企业上市工作，年内力争再有新的企业上市。鼓励项目主体通过股份转让、产权抵押、预期收益转让等手段，自主筹措建设资金，积极引导从煤炭资源整合中剥离出的资金投向“343”项目建设。

严要求推进项目建设。坚持把项目建设作为保持增长的主要载体和改善民生的直接抓手。突出大项目优先原则，对投资额大、带动力强，有利于调结构、促转型的大项目、好项目，在工作中倾斜扶持，在考核中提高赋分权重，并实行包联责任制，全力以赴加快推进。加大审批攻坚力度，围绕立项、环评、用地三大环节，在严格审批程序、严防违规建设的前提下，主动到县预审、市级集中会审、行业分解助审、全力以赴突审，争取项目早日获批。全面执行工程规范化管理，不断完善质量监管、工程监理和责任追溯制度，确保施工、生产安全。严格考核奖惩兑现，将项目推进情况纳入年度目标责任制考核范畴，依据评分标准对各县市、区 打分排队，按照工作职责对市直相关部门评估考核。对责任不落实、工作不到位、任务完不成的，或因人为因素导致工程质量低下、发生安全事故的，实施严格的行政问责。要超前谋划，制定科学的水、电、运力等要素配置方案，保障项目建成之后的运行需求，力求早投产、早受益。

大力度抓好旗舰项目。突出重点，集中力量抓好35个投资过10亿元以上的在建项目。年内建成兴县斜沟1500万吨、临县锦源600万吨、中阳荣欣300万吨、汾阳龙峰120万吨矿井等一批煤炭提升项目，建成柳林联盛360万吨水泥、交城宏特10万吨针状焦等一批工业转型项目，建成太中银铁路、太佳高速公路、汾平高速公路、340省道改造等一批基础设施建设项目。力争用3—5年的时间，全面实现我市煤焦、冶金龙头产业集群利税超100亿元、一批重点企业产值超100亿元的“双百亿”创建目标，构建起支撑我市产业转型、经济跨越、整体崛起的基础设施条件和核心产业体系，让“富裕吕梁”具有更加扎实的基础。

2、切实加强“三农”工作，促进农业增效农民增收农村繁荣

全面落实强农惠农政策。认真兑现国家粮食直补、良种补贴、农机具购置补贴、农资综合补贴等政策措施。不断提高“三农”投入比例，新增基础设施和社会事业发展投入60％以上用于农村，土地出让收入80％以上用于农业和农村基础设施建设，新增耕地占用税收入、新增建设用地土地有偿使用费全部用于农业发展。创新“以工补农”、“以煤促农”制度体系，构建输血与造血互补、扶贫与富民互动的长效机制。

继续加强农业基础建设。实施耕地综合生产能力建设、中低产田改造、旱作节水农业“三大工程”，加快建设一批新水源、水保生态和提黄灌溉项目，年内建设淤地坝60座，新增基本农田2．7万亩，完成水保初治面积40万亩，生态修复面积80万亩。扶持发展农机大户、农机合作社和专业服务组织，提高农业机械化服务水平。抓好科技示范展示、新型农民培训、基层农技推广体系建设与农产品质量安全认证“四项工程”，提高农业科技含量。提高异常气候应对能力，加强农业防灾减灾工作。

积极发展特色现代农业。按照“一村一品、一乡一业、一县一特”的发展模式，着力推进杂粮、畜牧、林果、蔬菜、劳务等特色产业规模化发展。深入推进“三大经济林覆盖”工程，抓好200万亩玉米增粮战略以及300万亩杂粮、30万亩玉米丰产方建设。继续抓好雁门关生态畜牧经济区建设，年内发展100个规模健康养殖小区。大力推广马铃薯高产技术，继续实施菜篮子“市长工程”，加快建设中部与平川两大蔬菜基地。扶持五大现代农业园区向全省一流示范园区迈进。积极推进农产品加工“513”工程，支持龙头企业做大做强，年内新增2个销售收入超亿元的龙头企业，5－8个超5000万元的龙头企业。大力推动劳动力异地输出就业，发展特色劳务经济，打造吕梁劳务输出品牌。

不断深化农村综合改革。稳步推进农村土地经营制度改革，探索建立土地流转市场，鼓励农民依法自愿有偿流转承包土地。扎实推进供销社各项改革。积极发展多种形式的适度规模经营，提高农业集约化、组织化水平。新发展农民专业合作社400个。深化农村金融改革，积极发展村镇银行、小额信贷公司、农村资金互助社等新型农村金融组织，三年内消除金融机构空白乡镇。开展政策性农业保险试点工作。

进一步加快扶贫开发进程。不断加大贫困村“三个确保”扶持力度，突出“低保对象”与“低收入对象”的政策衔接，把扶贫工作目标从减少贫困人口数量向增加贫困人口收入调整，不断提高扶贫开发水平。继续实施整村推进、移民扶贫、转移培训、产业扶贫、教育扶贫五大扶贫工程。年内再解决11．6万绝对贫困人口的温饱问题，让更多的贫困群众能吃饱穿暖，过上安康日子。

3、深入实施产业调整振兴规划，加快市域经济转型发展步伐

继续帮扶优势骨干企业。启动“方阵”扶持措施，支持第一方阵年销售收入30亿元以上的传统产业企业、5亿元以上的新兴产业企业和纳税5亿元以上的企业，实施跨地区、跨行业、跨所有制兼并重组，积极承接和参与国内外产业分工；支持第二方阵年销售收入10亿元以上的传统企业、1亿元以上的新兴企业和纳税1亿元以上的企业，建立完善现代企业制度，加强技术改造，转变管理模式，延伸产业链条，增强抵御市场风险能力；对第三方阵年销售收入5亿元以上的传统企业、5000万元以上的新兴企业和纳税5000万元以上的企业，实行跟踪服务，引导其及时调整发展方向，加快建设速度，抢占产业和市场制高点。

积极支持民营经济发展。支持民营企业通过合资、合作、托管等多种形式，大力兴办科技开发、信息工程、技术咨询、技能培训等项目。扶持创办利用农业农村资源的民营中小企业，帮助更多农民从传统耕作中转移出来。降低准入门槛，鼓励民间资本以独资、参股、特许经营等多种形式进入第三产业和公用基础设施领域。认真落实税费改革等政策措施，加大土地供应、税收优惠和财政贴息扶持力度，降低民营企业特别是新兴产业领域的中小企业运行成本。

全面实施产业调整振兴规划。煤炭行业，继续抓好煤矿兼并重组整合，加快企业证照办理、手续完善、矿井改造提升和复工复产步伐，鼓励主体企业加快发展循环经济，促进企业生产步入规模化、集约化、机械化、信息化轨道。冶金行业，围绕“双千万吨级”吕梁钢铁基地建设目标，加快太钢岚县项目建设，支持中钢、海威钢铁集团兼并联合；加快交口、兴县、孝义等地氧化铝项目建设，提高行业集中度和竞争力。焦化行业，按照“控制总量、置换提升、联合重组、做大做强”的思路，改造和新建一批炭化室高度在7．3米以上的项目，着力构建以孝义梧桐、离石信义、交城夏家营、汾阳三泉、中阳金罗等园区为重点的焦化生产基地。煤化工行业，大力发展焦油加工、粗苯精制、碳化工、甲醇烯烃化、化肥、钾盐、二硫化碳等七大特色产品，打造具有吕梁特色的煤化工基地。电力行业，以“变输煤为输电、构建环网架构”为方向，加快重点电源项目的立项及开工建设步伐，尽快开工吕梁2座500千伏输变电工程及吕梁至稷山500千伏线路等重点项目。装备制造行业，培育以矿用设备、汽车配件、铁路轨道配件为主导的机械加工制造产业集群，打造以城市建筑管盖、大型铸管、汽车铸件、石油钻杆为主导的铸造产业集群，着力培育10户以上销售收入超5亿元的龙头企业。新型材料行业，以福龙、吉港公司为龙头，大力推动水泥基地建设，着力构建玻璃墙材、阻燃耐材两个产业集群，形成门类齐全、布局合理的产业发展格局。食品行业，把杏花村酒业集中发展区建设作为全市产业转型的“一号工程”，放大汾酒的品牌效应，着力推动区域白酒资源整合，努力实现“35311”建设目标用3年时间，投资50亿元，新建酒业集中发展区，安排3万人就业，带动10万高粱种植户致富，销售收入达到100亿元以上 。支持以红枣、核桃、沙棘、肉类特色食品、保健品、果品、果蔬、食用油八大系列产品为主导的产业群建设。适应新能源产业发展形势，尽快上马一批风能发电和天然气、煤层气综合利用项目，推动新能源产业加快发展。深入实施服务业发展“1＋10”工程，促进第三产业健康快速发展。

深入推进产业布局调整。积极推进离石、中阳、柳林、方山中部四县区 的战略协作，以一批煤炭、焦化、冶金、化工、物流、旅游和现代服务业项目为载体，加快培育“离石经济圈”；充分利用平川四县市 经济技术、基础设施、地域文化等优势，借力“太原经济圈”，着力建设“平川经济片”；积极支持太钢、中铝、霍电、美锦、华润联盛等企业的特大型项目建设，带动县域经济跨越崛起，努力打造南北五县“新兴增长极”。通过各区域梯次发展、错位发展、竞相发展，促进后发展地区与先发展地区一道走上统筹协调可持续的科学发展之路。

4、大力推进节能减排和生态建设，探索低碳化发展路径

深入开展环境质量提升年活动。全面启动环境污染巡查整治、工业污染源水（气）排放口规范化整治、城市环境基础设施建设督查、城乡重点部位环境卫生综合整治与城市悬浮颗粒物整治、重点流域巡查整治、主要交通干线环境建设与整治、土小及污染严重企业清理、煤炭开采生态环境恢复治理等“八项行动”。认真执行《规划环境影响评价条例》，严格落实“三同时”制度，重点完善各类工业园区、工业集中区的区域规划环评工作。继续实施蓝天碧水工程，加大重点企业、设施的监管力度，全面推进高速沿线和重点河流沿线企业排污口规范化整治，确保重点区域工业污染源水气排污口规范化整治率达到100％。严格执行断面水质考核生态补偿机制，强力推进城市污水垃圾集中处理、集中供热为重点的城市环境基础设施建设，确保年内县县建成生活污水处理厂，全面实现“十一五”减排目标。

切实加大节能降耗力度。强化对各县市、区 政府和153户重点用能企业的节能目标责任的评价考核。继续实施节能重点工程，全面淘汰不符合产业政策的焦化、钢铁、小水泥、小造纸等落后产能和装备，坚决取缔无证水泥企业，先行禁止离石、汾阳、孝义城区使用实心粘土砖，大力推广新型墙体材料。普及推广先进节水技术，积极推进矿井废水利用和中水回用，单位产出取水量再降8％。认真执行项目节能评估和审查制度，未通过审查的项目一律不予立项，不符合政策的项目一律叫停。加大市场引导和行政推动力度，加强建筑、交通运输、公共机构、商贸服务等重点领域节能工作。

继续实施造林绿化工程。围绕扩大新造林、管好现有林、巩固退耕林、保护生态林、壮大干果林、提升景观林、开发旅游林的总目标，继续推进通道绿化、平原农田林网绿化、重点景区绿化、经济林覆盖、城郊森林公园、矿区植被恢复六大造林绿化工程，加快构建富有吕梁特色的森林生态体系、林业产业体系、森林文化体系。全年新增造林面积60万亩，林木覆盖率提高1．5个百分点。稳步推进集体林权制度改革，力争全市林改面积达到500万亩以上。积极实施碳汇造林项目，提高碳汇基金使用效益。

稳步推进生态环境治理修复。以打造吕梁的花园、城市的绿肺为目标，实施草地治理、河道整治、沿岸绿化等项目，加快汾河、三川河等重点流域生态环境治理修复步伐，全面完成年度建设任务。推进其他重点流域的治理修复工作，加强水源地保护，加快文水、方山省级湿地公园建设，保护好吕梁“城市之肾”。在“十一五”规划的交账之年，我们有信心向省委、省政府交出一份满意的答卷，也有责任让全市人民的生活环境有一个新的改善！

5、着力提高城市建设与管理水平，加快城乡统筹发展进程

搞好城市规划和建设。做好吕梁市区总规修编、控规完善和专业规划、修建性详规以及县（市）域村镇体系规划、重点建制镇规划、名镇名村保护规划的编制工作。研究制定离（石）柳（林）中（阳）方（山）城市群规划，争取进入全省统一规划。继续坚持市区“北拓、东延、南进”的思路，统筹旧城改造与新区开发，以机场大道、环城高速、文化广场、火车站站前广场、集中供热系统完善工程等重点市政基础设施建设工程为抓手，完善功能，培育产业，提高城市辐射带动能力。鼓励其他县市 以城市和县城建设为龙头，以重点建制镇提升为支撑，发挥比较优势，科学规划布局，完善设施，改善面貌，统筹推进特色城镇化进程。坚持与时俱进，精心打造一批标志性建筑和亮点工程；注重挖掘历史文化内涵，加快建设一批具有地域特色的街区、村镇、景区，不断提升全市城镇化水平。

加强城市管理和城乡环境整治。严格执行《山西省城乡规划条例》，切实维护各项规划的法定效力，严肃项目审批，严格监察督导，严查违规建筑，严厉追究责任。积极探索适合我市实际的户籍制度改革和管理模式，有序吸纳农村人口来城市和县城就业、就学和居住。坚持“以人为本、服务至上”的理念，进一步改进城市管理方式，让广大市民充分享有高水平的管理和人性化的服务，体验宜居城市的魅力。按照“一年初见成效、三年显著改善”的要求，以市区县城 规划区、城乡结合部、重要集镇、国省干道和县乡公路两侧、景区景点、工业园区周边等关键部位为重点，大力开展城市和农村环境综合整治工程，集中整治乱扔乱倒、乱搭乱建、乱停乱放顽疾，尽快解决“脏、乱、差”问题。深入开展市民素质提升行动，采取教育、引导、强制等措施，增强“人人都是软环境”的意识，努力创建清洁文明、和谐宜居的城乡环境。

加快特色城镇和新农村建设。在布局重大工业项目和规划工业园区时，超前制定城镇发展规划，同步建设城镇公用设施，力求实现“投产一批大项目，提升一个小城镇；建设一个城镇群，隆起一条经济带”的良性循环。坚持新农村建设与特色城镇建设并重，进一步完善项目整合、资金捆绑、企业帮建、单位帮扶四项机制，抓好新启动的228个重点村建设。鼓励经济基础较好的县市、区 加速推进村镇一体化，支持基础相对较差的县量力而行，稳步推进。全面启动新农村建设“百村行动计划”，每个县市、区 每年建成5—10个高标准新农村，力争通过3—5年的努力，让全市农村面貌发生根本改观，充分展现吕梁新世纪新农村、新时代新农民的新形象！

6、积极推动社会事业发展，提高基本公共服务均等化水平

优先发展教育事业。大力实施校舍安全工程，全面完成校舍安全“全覆盖”任务。落实义务教育经费保障机制，稳步推进义务教育标准化建设。优化师资队伍区域化布局，落实国家农村特岗教师计划，开展送课下乡、对口支教等工作，促进城乡教育均衡发展。加快布局调整，深化课程改革，推动普通高中向办学规模化、管理精细化、硬件标准化目标迈进，鼓励条件成熟的县市、区 率先普及高中阶段教育。重视学前教育，全面启动“一个乡镇一所示范幼儿园”建设工程。加快吕梁学院、吕梁职业技术学院建设与发展，推进中等职业教育资源整合，支持师范教育健康发展，为全市经济社会发展培养更多专业人才。实施市区教育振兴工程，扩大办学规模，提升办学水平，借助优质教育资源集聚人口、增强综合竞争力。广泛开展全民健身活动，努力提高竞技体育水平。

大力发展卫生计生事业。落实国家基本药物制度，推进公立医院改革试点，全面开展“办人民满意卫生、建人民满意医院”活动，不断提高医疗质量和服务水平。认真组织实施基本公共卫生服务项目，稳步推进基本卫生服务均等化。加强基层医疗卫生机构建设，努力提高村卫生所“全覆盖”标准。巩固扩大新型农村合作医疗工作成果，确保参合率稳定在90％以上。深入推进中医药振兴工程，支持中医院建设，发展中医事业。积极开展重大传染病防治工作，努力提高应对突发公共卫生事件的能力。落实长效节育措施，实施优生促进工程，稳定低生育水平，提高人口素质。健全食品药品监管体系，开展食品药品专项整治，确保人民群众吃上放心食品，用上放心药品。

切实加强科技与人力资源建设。加快科技创新体系建设，开展新型工业、现代农业技术集成推广活动，以企业和科研院所为主体，组建技术创新联盟，努力在能源化工、新型材料等关键领域和采煤设备研制、红枣防裂果等重点项目上取得突破。积极营造有利于科技创新的社会环境，在全社会形成尊重劳动、尊重知识、尊重人才、尊重创造的良好氛围。牢固树立“人力资源是第一资源”的理念，积极探索和加快构建人力资本与项目相结合的引才渠道、用才机制和留才途径，努力为各类人才充分展示才华搭建平台。

加快发展文化旅游等社会事业。继续深化文化体制改革，大力发展新闻出版、广播影视、文学艺术等公共事业，不断丰富人民群众的精神文化生活。深度挖掘和加快发展黄土黄河文化、革命传统文化、古建民居文化、民间民俗文化，推出一批文化艺术精品，促进文化产业繁荣。制定和实施吕梁旅游发展总体规划，启动交城、文水、汾阳、离石、方山联合开发庞泉沟（关帝山）生态旅游度假区等重点旅游项目，打造一批精品线路，加快旅游产业发展。支持工会、共青团、妇联、科协依照章程开展工作，关心老龄、残疾人事业发展，做好外事侨务、对台事务、民族宗教、档案史志、防震减灾、人民防空等工作。支持国防建设，做好双拥工作，促进军民融合式发展。总之，我们将不断加大投入和工作力度，努力使各项社会事业的发展与经济增长同步、与时代潮流同步、与人民群众多样化需求同步！

7、更加注重保障和改善民生，开创和谐社会建设新局面

深入实施“九个全覆盖”工程。对照省政府下达的“五个全覆盖”目标任务，强化工作措施，加快推进步伐，在全面完成年度建设任务的同时加强业务管理，提升服务质量，实现动态“消零”，做到硬件、服务“双覆盖”。加快我市自主确定的城乡困难群众最低生活保障全覆盖、城乡困难群众大病医疗救助全覆盖、中小学家庭经济困难学生资助政策全覆盖、农民和缺乏基本医疗保障的城镇居民合作医疗全覆盖等“四个全覆盖”的政策衔接和工作推进步伐，确保取得实效，绝不失信于民！

不断完善就业社保体系。落实就业创业优惠政策，鼓励大中专毕业生到基层、中小企业就业或自主创业，帮助“4050”人员、残疾人、下岗职工等困难群体就业。促进农村劳动力就业转移。完善社会保障体系，扩大新型农村养老保险试点，推进事业单位养老保险制度改革，进一步扩大农民工、个体工商户、民营企业职工参保覆盖范围，社会保险综合覆盖率达到85％以上。提高城乡低保、农村五保人员、优抚对象的保障补助水平。大力发展社会福利及慈善事业，积极开展救灾救济工作。加快城乡保障性住房建设，着力解决困难群众住房难题，有条件的县（市、区）可探索公租房建设模式，尽可能降低外来技术人才、新就业大中专学生和进城务工人员的住房门槛。

切实加强安全生产工作。前不久发生的王家岭煤矿透水事故和近年来吕梁发生的一系列安全事故充分表明，安全生产工作不可能毕其功于一役。巩固和发展我市目前来之不易的好局面，必须做到警钟长鸣，常抓不懈。突出煤矿安全生产这一重中之重，充分发挥煤矿安全管理“六大员”和“五人”监管小组的作用，认真执行对复工复产、过渡期生产、应关未关矿井的分级监管制度，继续落实“一通三防”措施，扎实开展瓦斯、水患排查整治，认真实施在建和改扩建项目专项整治，严厉打击私挖滥采和违法违规生产行为，严防重特大煤矿事故发生。深入开展“安全生产年”活动，认真组织各行业、各领域的安全生产大检查。加大安全投入，强化人员培训，不落实安全投入的企业不许生产，不经过安全培训的人员严禁上岗。实行领导干部“一岗双责”制度，落实政府、企业两个主体和部门监管责任，建立事故问责和隐患问责相结合、追究企业责任和追究监管人员责任相结合的责任追究体系，前移安全责任追究关口，加大事故责任追究力度，切实营造安全生产是“高压线”的氛围。继续强化非煤矿山安全监管，严格执行道路交通安全事故行政问责制，进一步加强黄土高原地质灾害、尾矿库、人员密集场所消防、建筑施工、民爆物品、危险化学品等重点行业领域的专项整治，遏制重特大事故，压减一般事故，开创“安全吕梁”新局面。

努力提高社会管理水平。坚决纠正征地拆迁、企业改制、环境保护、劳动争议、涉法涉诉等领域损害群众利益的突出问题，切实保障人民群众合法权益。建立完善各种预警机制，畅通信息渠道，提高发现、研判和控制矛盾的能力，制定预案，前置防范和化解矛盾的防线，尽可能把不稳定因素消除在萌芽状态。充分发挥人民信访、民政协调、法律救助等制度的作用，引导群众以理性合法的形式表达利益诉求，解决纠纷问题。建立健全应急管理和救援体系，进一步增强处置突发事件的能力。加强社会治安综合治理，健全治安防控体系，防范和依法打击各类违法犯罪活动，为全市人民打造一个安全和谐的舒心家园！

办好让多数群众长远受益的实事，是广大群众对我们的期望，也是人民政府的责任。今年，我们将继续为全市人民办好以下十件实事：

一是全面完成17．1万平方米的中小学校舍安全改造任务，把学校建成最安全、学生家长最放心的地方。二是在8个县（市、区）实行基本药物制度及药品零差率销售，完成4个县医院、3个中心卫生院、2个社区卫生服务中心的改扩建任务。三是新农保试点扩大到13个县（市、区），覆盖人数达到40万人；对1759名就业困难人员实施就业援助。四是新建、改扩建农村敬老院13所，为2000名贫困残疾儿童实施康复救助。五是新建廉租住房2590套，经济适用房1525套，城市棚户区改造510套，国有工矿棚户区改造233套。六是再解决43．36万农村人口的安全饮水问题。七是为295个建制村新建水泥（油）路1620公里。八是新建155家连锁农家店便民店 ，建设改造市级放心粮油配送中心1个、县级配送中心13个，示范店160个、经销网点1300个。九是新建移动大棚1500亩，新建和改造日光温室1500亩，新转移农村劳动力5万人。十是完成1570个自然村广播电视全覆盖任务。

各位代表，保持经济平稳较快发展、转变经济发展方式，必须要有切实的抓手和具体的支撑。从吕梁目前的实际出发，我们将结合“十二五”规划的编制和实施，在今后的工作中突出抓好以下四个关键环节：一是紧紧扭住项目建设不松手，不动摇；二是以经济开发区和工农业园区为载体，高效利用基础设施和发展要素，集约发展，循环发展；三是强化科技创新，讲适用，抓引进，不断提高核心竞争力；四是突出企业的主体作用，严格按照经济规律办事，尽最大努力支持各类市场主体，让一切财富源泉在吕梁大地充分涌流。为此，各级政府及其工作人员，一要加快转变发展观念。清醒认识转变经济发展方式的紧迫性，强化“永远吕梁，永久家园”的理念，决不以一时之利牺牲长远发展，决不为部分人的利益牺牲公共利益。坚持以绿色、循环、低碳为方向，以促进区域协调发展、城乡统筹发展为目标，科学规划产业布局，合理确定项目规模，不断提高经济增长的质量和水平。二要大力弘扬进取精神。处于承前启后、转型跨越关键时期的吕梁，在周边日趋激烈的发展竞争中不进则退、慢进如退。我们既不能满足于过去的成绩，也不能畏惧今后的困难，必须大力弘扬吕梁精神，自力更生，艰苦创业，求真务实，与时俱进，不断续写新时代的吕梁英雄传。三要继续加强行政效能建设。深入推进政府机构改革和事业单位分类改革，健全绩效评估机制，严格落实行政问责制，认真执行服务承诺、首问负责、限时办结、责任追究等制度，进一步加大政务督促、效能监察和审计监督力度，努力提高政府的公信力、执行力。四要切实改进工作作风。秉承发展为先、民生为重、实干为本的理念，深入开展“抓学风、正作风、树新风”活动，进一步精文简会，腾出更多的时间和精力学政策、搞调研、察民情、抓工作，杜绝不作为、乱作为，严查失职、渎职，努力建设人民满意的政府。五要认真落实廉政建设举措。继续加大对政府投资项目和公共资金的管理监督力度，切实纠正损害群众利益的突出问题。深入开展民主评议和督查整治，狠抓反腐倡廉制度创新和落实。引深煤焦领域、工程建设领域反腐败斗争，坚决查办违法违纪案件，不断推进政府系统廉政建设和反腐败斗争取得新成效。六要着力推进依法行政。自觉执行依法行政有关规定，促进公正执法、文明执法。完善公众参与、专家咨询论证和政府集体研究的决策机制，严格执行决策风险评估、失策责任追究制度，提高政府决策水平。自觉接受市人大及其常委会的法律和工作监督，主动接受市政协的民主监督，认真听取民主党派、工商联、无党派人士和各人民团体的意见，虚心接受舆论监督和社会监督，从善如流，广纳良策，努力开创全市上下群策群力抓工作，一心一意谋发展的生动局面。

各位代表，今日吕梁正处在转型发展、安全发展、和谐发展的历史新起点上。“雄关漫道真如铁，而今迈步从头越”。让我们在中共吕梁市委的坚强领导下，团结拼搏，开拓创新，奋发进取，扎实工作，为全面完成“十一五”规划的各项任务，不断开创“三新”吕梁的新局面而努力奋斗！
